# Assessment of a model for achieving competency in administration and scoring of the WAIS-IV in post-graduate psychology students

**DOI:** 10.3389/fpsyg.2015.00641

**Published:** 2015-05-18

**Authors:** Rachel M. Roberts, Melissa C. Davis

**Affiliations:** ^1^School of Psychology, The University of Adelaide, Adelaide, SAAustralia; ^2^School of Psychology and Speech Pathology, Curtin University, Bentley, WAAustralia

**Keywords:** training, professional psychology, teaching, intelligence, WAIS, post-graduate

## Abstract

There is a need for an evidence-based approach to training professional psychologists in the administration and scoring of standardized tests such as the Wechsler Adult Intelligence Scale (WAIS) due to substantial evidence that these tasks are associated with numerous errors that have the potential to significantly impact clients’ lives. Twenty three post-graduate psychology students underwent training in using the WAIS-IV according to a best-practice teaching model that involved didactic teaching, independent study of the test manual, and in-class practice with teacher supervision and feedback. Video recordings and test protocols from a role-played test administration were analyzed for errors according to a comprehensive checklist with self, peer, and faculty member reviews. 91.3% of students were rated as having demonstrated competency in administration and scoring. All students were found to make errors, with substantially more errors being detected by the faculty member than by self or peers. Across all subtests, the most frequent errors related to failure to deliver standardized instructions verbatim from the manual. The failure of peer and self-reviews to detect the majority of the errors suggests that novice feedback (self or peers) may be ineffective to eliminate errors and the use of more senior peers may be preferable. It is suggested that involving senior trainees, recent graduates and/or experienced practitioners in the training of post-graduate students may have benefits for both parties, promoting a peer-learning and continuous professional development approach to the development and maintenance of skills in psychological assessment.

## Introduction

Psychology as a profession is committed to developing and maintaining the professional competencies necessary for independent practice (Page and Stritzke, 2006) and attaining and maintaining competence is a fundamental component of ethical practice (Australian Psychological Society, 2007). Whilst clinical psychology training is based on the ‘scientist practitioner model’ and there is a strong emphasis on evidence-based practice, there is comparatively little investigation and evaluation of what might be considered ‘evidence-based teaching methods’ (Baillie et al., 2011). Specifically, whilst competency in the area of psychological assessment has been referred to as ‘a defining aspect of psychological expertise’ (Krishnamurthy et al., 2004, p. 726) and as an ‘essential area of competency for psychologists’ (Kuentzel et al., 2011, p. 39), there is a paucity of evidence on teaching and learning strategies for fundamental psychological assessment competencies such as the administration and scoring of standardized tests of intellectual functioning like the Wechsler Intelligence Scales (Wechsler, 2003, 2008, 2012).

Research evidence suggests that trainees as well as experienced practitioners typically make numerous errors in administering and scoring these tests; the number of errors made by trainees does not decrease with mere practice (e.g., Slate et al., 1991; Ryan and Schnakenberg-Ott, 2003; Loe et al., 2007) and the number or type of errors does not differ according to different editions of the tests (Kuentzel et al., 2011). Errors can be in administration (e.g., failing to follow the reverse-rule to establish a baseline, deviation from standardized instructions), scoring (e.g., incorrect allocation of scores to responses, errors in converting raw scores to scales scores) and/or computation (e.g., incorrect age calculation or arithmetic errors in summing scores; Belk et al., 2002). Hopwood and Richard (2005) noted that computer scoring programs should be used, however, Kuentzel et al. (2011) state that two thirds of errors made by their sample were calculation problems and would therefore not be corrected by using scoring software.

The impact of errors on test results is not insignificant. A study by Hopwood and Richard (2005) found that the Full Scale IQ (FSIQ) score was correct in only 41.7% of protocols, and in the Belk et al. (2002) study, 88% of protocols contained errors that affected the FSIQ. Errors may be due to carelessness (for example, failing to record responses) or difficulty understanding the scoring criteria. A number of authors (e.g., Fantuzzo et al., 1983; Belk et al., 2002; Brazelton, 2003; Ryan and Schnakenberg-Ott, 2003) have reported that errors are most frequent on the Verbal subtests (Vocabulary, Similarities, and Comprehension) due to failure to query marginal responses or incorrect assigning of points to ambiguous responses.

Error rates have been found to be unrelated to type of educational qualifications or time since completing degree (Ryan and Schnakenberg-Ott, 2003; Wolfe-Christensen and Callahan, 2008). Hopwood and Richard (2005) found that error rates did not decrease with experience and there was actually a positive correlation between self-rated competence and error rates. These findings highlight that the presumed ‘practice makes perfect’ model of competence in the use of the Wechsler Intelligence Scales is erroneous, with practitioners appearing to practice and reinforce their errors as well as drift from standardized administration procedures over time (Slate et al., 1991; Gilmore and Campbell, 2009).

Given the life-changing impact of intellectual functioning assessment results for clients, such as demonstrating that a client meets eligibility criteria for support services, research on error rates highlights the absolute importance of robust training in psychological assessment skills during formal education, including rapid, accurate feedback on errors to bring these into awareness and thus reduce the probability of incorporating these into one’s future practize. It is therefore apparent that an evidence-base of best-practice teaching strategies for Wechsler Intelligence Scale administration is required.

Blakey et al. (1987) found that trainees’ errors in scoring the Comprehension and Similarities subtests was significantly reduced following peer feedback via a checklist, but did not result in higher accuracy at the level of the total protocol. Kuentzel et al. (2011) reported a significant improvement in the percentage of error-free protocols following a peer-checking procedure (from 20 to 63%) and errors in the FSIQ were corrected in 66% of protocols. However, these results demonstrate that a simple checking procedure is insufficient to eliminate errors. Further, many errors cannot be detected from the completed protocol and this makes direct observation of administration an essential component of training (Gilmore and Campbell, 2009).

There are two known ‘models’ comprising a series of strategies aimed at promoting competent administration of the Wechsler scales. Fantuzzo et al’s. (1983) MASTERY model consists of (a) preliminary familiarization with the administration and scoring manual through 1–2 h study; (b) lecture on common administration errors; (c) observe three perfect administrations; (d) administer test three times with feedback. Fantuzzo et al. (1983) defined ‘competency’ as 90% accuracy on a checklist of 198 items. Despite the publication of these guidelines, there has been little empirical investigation of the effectiveness of these strategies and published studies have involved investigation of only one or some of the strategies For example, Fantuzzo et al. (1983) found that students significantly improved their accuracy of administration following watching an errorless administration with commentary about common errors and then being required to detect errors in another videotaped administration.

Blakey et al. (1987) conducted a controlled study of a peer-mediated version of the MASTERY model by comparing the performance of two groups of students. The control group was directed to study the test administration manual for a minimum of 2 h and then spend as much time as they needed familiarizing themselves with and practicing using the test materials. The experimental group underwent a systematic training procedure involving studying the administration manual for 2 h, achieving at least 90% on a test of the manual’s content, then participating in the roles of examiner, examinee and evaluator in a peer-review procedure involving practice administration of the test. The results of this study indicated that the peer-mediated MASTERY model was superior to study alone for reducing administration errors (*M* = 92.9% accuracy compared with *M* = 63.6% accuracy). Although there was no significant difference between the accuracy of scoring the whole protocol (*M* = 95.3% for the experimental group versus *M* = 92.6% for the control group), the experimental group scored the Comprehension and Similarities scales significantly more accurately.

Gilmore and Campbell’s (2009) model represents the most comprehensive published training model to date. The model is outlined in **Table 1** and addresses two key issues that have arisen in the literature; firstly, it aims to ensure that initial learning of the test administration and scoring is thorough and accurate so that students do not continue to practice errors in future experiences; and secondly, it encourages students to be highly self-aware and self-reflective in evaluating their own practice (Gilmore and Campbell, 2009). It also provides a model of peer-review and peer feedback that are important in professional practice (Gilmore and Campbell, 2009). The authors conclude that this training method is an affordable, competency-based training program but there is not yet for evidence of its effectiveness. Gilmore and Campbell (2009) acknowledge that their training method requires a very high time commitment from faculty. With a class size of 25, it is estimated that 107 h of faculty time is needed to deliver this method, a time commitment beyond what is practical in most Australian university settings. As such in the current study, the teaching model was modified to reduce the faculty time required, while maintaining key components (see **Table 1**).

**Table 1:** Training models for teaching and learning competent administration of the Wechsler scales.

Gilmore and Campbell’s (2009) model	Faculty teaching time (based on class size of 25)*	Modification of Gilmore and Campbell’s (2009) model for current study	Faculty teaching time (based on class size of 25)*
(a) Introductory didactic lectures		(a) Three 3-h seminars:	9 h
(b) Demonstration by faculty and supervised practice (c) Practice and peer feedback	14 h	(i) Introduction to the range of Wechsler scales, description of WAIS-IV structure, content, administration, scoring and reporting, common		
(d) Revision tutorial faculty observes administration	50 h	errors (ii) Assessment of intellectual ability in children		
(e) Videoed administration to volunteer		(iii) WAIS-IV practice with peers in class, with		
(f) Self, peer, and faculty review of video (g) Further practice	25 h	supervision and feedback (students study administration manual and familiarize themselves		
(h) Live administration of six subtests to faculty	25 h	with WAIS-IV prior to seminar) (b) Videoed administration of WAIS-IV to peer		
(i) Faculty review scoring and the requirement to repeat the assessment if failed	6 h	(c) Review peer’s video, and complete the Administration and Scoring Checklist for both themselves and peer		
		(d) Faculty review of video, students who did not demonstrate competency repeat assessment	50 h
	**107 h**		**59 h**	

*Based on estimated 2 h per review of Wechsler Scale video.

Given that there is a limited evidence base for teaching in this area, it is not surprising to see a variety of approaches used in Australian universities. Scott et al.’s (2011) survey of Clinical Psychology students and Clinical Psychology program directors revealed that although many students believe they are more competent to use testing materials when they have been taught in practical and interactive ways, most programs continue to teach students in didactic, exam-based styles. The authors state that traditional teaching methods do not have a current evidence base and there is a strong need for research to develop this evidence base. Gilmore and Campbell (2009) reported the results of their survey of training practices used by staff from 28 post-graduate psychology programs in Australia. They found that the most commonly reported teaching strategies were viewing a selection of subtests from a live or videorecorded test administration, having students read the administration manual, discussing administration issues, and practice test administrations. Staff cited restricted instruction time and availability of test resources as barriers to more comprehensive teaching of these skills.

Davis and Hollingworth (2012) conducted a survey of 12 academic staff who coordinated psychological assessment units in professional psychology training programs in Australian universities. They found that the teaching strategies most frequently rated as used ‘often’ were ‘information about common administration and scoring errors’ (100%), ‘didactic lectures’ (80%), and peer observation and feedback (80%). In contrast, the following strategies were infrequently rated as used ‘often’: ‘peer-checking scored protocols’ (20%), ‘video with self-review’ (20%), ‘observing ‘model’ administrations’ (10%), and video with peer review (0%). Interestingly, the majority of staff indicated that they believed that their teaching strategies represented ‘best practice.’

There is a need for empirical investigation of the effectiveness of training in the administration and scoring of the Wechsler Intelligence Scales, to guide the evidence-base to approaches to teaching competence in this key area. The aim of the current study is to evaluate the effectiveness of training post-graduate professional psychology students in two Masters programs in an Australian university. Training is based on Gilmore and Campbell’s (2009) best-practice model of training but modified to reduce the amount of faculty time required. Effectiveness is assessed in two ways; firstly by examining an overall rating of competency in the administration and scoring of the test, and secondly by examining error rates and types of errors following training. We also examine whether the errors identified by the faculty member were identified by either the students or peers.

## Materials and Methods

### Participants

Of the 23 post-graduate students whose work is reported here, 2 were male, 21 were female, and 18 were Master of Psychology (Clinical) Psychology students, and 5 were Master of Psychology (Health Psychology) students. Six students were concurrently enrolled in a Doctor of Philosophy (these students complete all coursework and placement requirements of the Masters program, as well as all requirements for a Ph.D. by research).

### Measures

#### Competency Rubric

Competency was assessed using a rubric. Students needed to demonstrate competency across all areas of administration and scoring to be rated as having demonstrated competency. Competency was judged in terms of whether the administration and scoring resulted in a valid assessment, that is, if this was an assessment of a real client, could the results be used? Errors such as not recording client responses verbatim (e.g., omitting a word that didn’t impact on the score), or not recording time taken on sample items, which were very unlikely to have impacted on the validity of the administration or score, were not considered sufficient to judge the administration as invalid. Hence, if a student only made a small number of minor errors, they would have been judged as having demonstrated competency. Errors such as incorrect totalling of scores (such as not including the scores for the items before the start point), or not reading instructions verbatim (summarizing instructions in the student’s own words for instance) were considered to have impacted on the validity of the assessment and resulted in the student not being rated as having demonstrated competency. For more details, see the copy of the assessment rubric in Supplementary Material 1.

#### Administration and Scoring Checklist

The Administration and Scoring Checklist used in the study was developed by the second author and has been used for several years in the Psychological Assessment unit in the Master of Psychology programs at Curtin University. Previous checklists (e.g., Fantazzo and Moon, 1984; Blakely et al., 1985; Sattler, 2008, cited in Gilmore and Campbell, 2009) were considered unsuitable for the current study as they included subjective criteria that are not indices of ‘accuracy’ but rather relate to other qualities of the administration such as organization of materials, sensitivity to the examinee’s need for a break, and quality of the rapport between the examiner and examinee. Further, these checklists were developed on outdated editions of the Wechsler Scales, hence a checklist that was based on the administration and scoring criteria of the current edition (WAIS-IV) was required.

The Checklist was developed by extracting all the essential administration, scoring, and score conversion criteria from the WAIS-IV Administration and Scoring Manual (Wechsler, 2012). As such, the checklist was constructed in two parts. The first part contains a set of criteria related to Administration and Subtest scoring, covering a total of 117 points (6–13 criteria per subtest). Some criteria were scored from the video recording of the administration (e.g., accuracy of general directions, use of queries if necessary) and others were scored from the completed protocol [e.g., reverse rule (if necessary), discontinuation rule, recording of responses, item scoring, total raw score]. Each item was scored as either Satisfactory/Correct or Unsatisfactory/Incorrect and the reviewer could also make a qualitative comment for each.

The second part covered criteria related to Score Conversion and Process Analysis. Sixteen items were rated as either ‘correct’ or ‘incorrect’ and could be accompanied by a qualitative comment. The items were: age calculation, scaled scores for each subtest, sums of scaled scores for each Index and the FSIQ, IQ/Index scores, discrepancy comparisons, strengths and weaknesses analysis, process analysis, and appropriate use of substitute subtest/s. A full copy of the Administration and Scoring Checklist is provided in Supplementary Material 2.

### Procedure

This research project was approved by the University of Adelaide, School of Psychology Human Ethics Subcommittee. The University of Adelaide 2013 class enrolled in the Psychological Assessment course were invited to give consent for their assignment material to be used for this research project (*n* = 23 gave consent, *n* = 3 did not give consent). Students take this required course in their first semester of graduate study. The teaching and learning model is summarized in **Table 1**. Course content relevant to the assessment of intelligence (within the 12 seminar course) included three 3-h seminars. Students were then required to video record one administration of the WAIS-IV to a classmate, to review the classmate’s video, and complete the Administration and Scoring Checklist for both themselves and their peer. Students were encouraged not to submit their video until they were confident that the assessment and scoring was valid. Finally, the video recording was reviewed by a faculty member (first author), the checklist completed, and competency assessed. Students also completed a report of the test results which was marked separately. Students were provided with the faculty member’s copy of the checklist. Additional specific feedback was also provided if other issues arise which were not captured in the checklist. Students who did not demonstrate competency were required to submit another recording.

In summary, three Administration and Scoring Checklists were completed, one by the faculty member, one by the student about themselves, and one by a peer (another student). Of note, when students completed the Administration and Scoring Checklist, either in relation to their own performance or that of a fellow student, they sometimes described in the comments section the details of an error, but then indicated that the aspect being rated was ‘correct’ rather than ‘incorrect.’ In these instances, this was not recorded as an identified error as the student did not indicate that they appreciated that this was a significant error in administration or scoring.

## Results

### Competency

Each student’s competency in administration and scoring of the WAIS-IV was determined according to the faculty member’s review. Overall, 21 of the 23 students (91.3%) were assessed as competent. Two students made errors in administration or scoring which were sufficient to alter the test results (the IQ scores obtained) and so, after individual feedback, these studentssignment (both demonstrated competency on their second attempt).

### Administration and Scoring Errors

The following results are based on the faculty member’s review on the Administration and Scoring Checklist although comments are made about the consistency between errors detected by self, peer, and the faculty member. Every student made one or more errors. Of the 117 rated aspects of administration and scoring, no errors were made by any student on 59 of the areas assessed. Aspects of the administration and scoring that were error-free in at least some subtests were; use of start, reverse and discontinuation rules, item recording and scoring of most subtests, as well as score calculations and the age calculation. Overall across all subtests, 176 errors were identified by the faculty member, 21 errors were self-identified, and nine by peers. While self and peer identified errors were far fewer than those identified by the faculty member, they were in most cases in the same areas. In the few cases where they were not, this was because the faculty member did not consider that an error had been made, or it was best categorized under a different item. In total, 93.5% of the faculty member-rated items were correct, with similar rates across the four Indexes (VCI 93.3%, PRI 95.7, WMI 91.8%, PSI 94.7% correct).

The number of errors detected by the faculty member, self, and peer for each subtest are presented by Index score. Errors identified in the Verbal Comprehension Index subtests are reported in **Table 2**. Common errors included the general directions not given verbatim from the manual, not querying responses that should have been queried, not recording responses verbatim, and incorrect item scoring.

**Table 2:** Faculty member, self, and peer ratings of errors Verbal Comprehension Index subtests.

Subtest error item	Faculty member identified (*n*)	Self identified (*n*)	Peer identified (*n*)
**Similarities**
(1) General directions	3		
(2) Use of queries	3	1	3
(5) Record responses verbatim	4		
(6) Item scoring	3	1	
**Vocabulary**
(1) General directions	2		
(2) Use of queries	2	2	1
(4) Discontinuation rule	1		
(5) Record responses verbatim	1		
(6) Item scoring	3		
**Information**
(1) General directions	4	1	
(2) Use of queries	1		
**Comprehension**
(2) Use of queries	9	2	1
(4) Discontinuation rule	2		
(5) Record responses	2		
(6) Item scoring	3		
**Total**	**43**	**7**	**5**

Errors identified in the Perceptual Reasoning Index subtests are reported in **Table 3**. The most common error was in the general directions not being given verbatim from the manual, although in many cases this involved minor omissions. Other common errors were in the layout of blocks in Block Design, not recording completion time (usually for sample items), and in Picture Completion common errors were not querying responses, and not recording whether the examinee had pointed or given a verbal response.

**Table 3:** Faculty member, self, and peer ratings of errors Perceptual Reasoning Index subtests.

Subtest error item	Faculty member identified	Self identified	Peer identified
**Block design**		
(1) Introduce blocks 5		
(2) Demonstration item	6	1	
(3) Lay-out of blocks	3		
(5) Scrambling blocks between trials	1		
(9) Record completion time	1		
(10) Draw incorrect design	2	1	
(11) Indicate whether correct design	3		
**Matrix reasoning**		
(1) General directions 4		
(2) Item instructions	2		
(3) Reverse rule	1	1	
(5) Record responses	1		
**Visual puzzles**			
(1) General directions	8		
(5) Record completion time	1		
**Figure weights**		
(1) General directions 7		
(2) Timing	1		
(3) Reverse rule	1		
(5) Record completion time	1		
**Picture completion**		
(1) General directions 7		
(2) Use of queries	4		
(6) Record responses (verbal and/or point)	5	1	
	**64**	**4**	**0**

Errors identified in the Working Memory Index subtests are reported in **Table 4**. The most common error was not delivering the Digit Span item at 1 digit per second. Other common errors were in the instructions not being given verbatim from the manual, with the majority of these errors being minor omissions.

**Table 4:** Faculty member, self, and peer ratings of errors Working Memory Index subtests.

Subtest error item	Faculty member identified	Self identified	Peer identified
**Digit span**		
(1) Item instructions 7		
(2) Pace of digits (1 per second)	8	2	
(4) Record responses		1	
(6) Total raw score	1	1	1
**Arithmetic**		
(1) General directions 7		
(2) Timing	1		
(4) Discontinuation rule	1		
(5) Record completion time	1		
(7) Item scoring		1	
**Letter-number sequencing**		
(1) Item instructions 6	1	
(2) Sample items	5	1	2
(3) Corrections/prompts	1	1	
(5) Record responses	1	1	1
(6) Item score	1		
	**40**	**9**	**4**

Errors identified in the Processing Speed Index subtests are reported in **Table 5**. The most common error was not delivering the instructions verbatim from the manual, again with most errors being minor omissions.

**Table 5:** Faculty member, self, and peer ratings of errors Processing Speed Index subtests.

Subtest error item	Faculty member identified	Self identified	Peer identified
**Symbol search**		
(1) General directions 2		
(2) Item instructions	6		
(3) Sample items	6		
(4) Practice items	2		
**Coding**		
(1) General directions 2		
(2) Item instructions	5		
(3) Timing	1	1	
**Cancelation**		
(1) General directions 3		
(2) Demonstration item	1		
(3) Sample item	1		
	**29**	**1**	**0**

The score conversion and process analysis was also assessed, with students overall making only two errors in these areas. One student made an error in calculating the Perceptual Reasoning Scaled Score, and one made an error in the process analysis, with the error identified by the student and peer.

## Discussion

This study aimed to provide a preliminary assessment of the effectiveness of teaching strategies based on the Gilmore and Campbell (2009) model for facilitating competence in the administration and scoring of the Wechsler Intelligence Scales (specifically the WAIS-IV) in post-graduate professional psychology students. Student competency was evaluated, and a triangulated process of self-review, peer-review, and faculty-marking allowed for an examination of the patterns of errors across the various components of the assessment as well as an analysis of discrepancies between the evaluations of different reviewers.

Consistent with the literature, all students made errors in one or more aspects of the administration or scoring. The majority of students were assessed to have demonstrated competency (91.3%). However, two of the 23 students were assessed by the faculty member to make errors that were substantial enough to affect the validity of the assessment and change the FSIQ score. This figure is substantially lower than error rates reported in the literature (e.g., Belk et al., 2002; Hopwood and Richard, 2005). However, students were encouraged not to submit their video until they were confident that the assessment and scoring was valid. This means error rates in the current study are likely to be a significant underestimate of the proportion of initial administrations that contained substantial errors as anecdotal feedback from students was that they typically re-recorded their administration if they or their peer detected significant errors.

Across all of the Index score areas, the most common error was the failure to deliver the general directions and item instructions verbatim from the administration manual. It is notable that this error was very rarely detected in the self or peer reviews. This may have been due to a lenient interpretation of ‘verbatim’ due to insufficient awareness of what level of ‘verbatim’ is expected and required for standardized administration. This suggests that initial education about standardized administration should emphasize the importance of verbatim adherence to manualized instructions and possibly include live or videorecorded demonstrations of this in practice.

Within the Verbal Comprehension subtests, use of queries and item scoring were the areas in which the most errors were detected by the faculty member. These errors were rarely detected in the self or peer review. These errors are consistent with findings in the literature of the frequency of errors related to querying marginal responses and scoring verbal subtests items (e.g., Fantuzzo et al., 1983; Belk et al., 2002; Brazelton, 2003; Ryan and Schnakenberg-Ott, 2003). Difficulties in these areas suggest that more extensive practice with evaluating responses to verbal subtests during training may be beneficial. A high level of familiarity with the general scoring principles and the specific exemplars for 2-point, 1-point, and 0-point responses is required in order for trainees to be able to make accurate decisions about responses during the test administration, whilst maintaining rapport with the examinee and maintaining the flow of the administration. In addition to previously mentioned strategies, practice strategies that may be useful in this regard include scoring hypothetical or de-identified protocols, students identifying errors in sample protocols, and peer role play (Davis and Hollingworth, 2012).

The Perceptual Reasoning Index subtest with the greatest number of errors (as well as the greatest number of criteria for potential errors) was Block Design. Again, the results showed that errors were rarely detected in peer and self-reviews. The relatively high rates of errors is consistent with previous findings that the practical aspects of the Block Design subtest administration adds significant complexity to the task, resulting in many errors, even following training (e.g., Moon et al., 1991). This suggests that particular attention needs to be paid to demonstrating accurate administration and students practicing under supervision until perfect administration can be achieved.

Working Memory Index subtests require accurate adherence to standardized instructions as well as careful progression through sample items. Whilst there were some errors in these aspects of administration another source of error, consistent with the most frequent error reported by Moon et al. (1991), was the failure to deliver the Digit Span items at the required rate of one per second. Errors can involve ‘chunking’ (items not being administered at a consistent rate) and/or the pace of delivery being too fast or too slow, all of which can significant impact the examinee’s performance. It is important that specific attention be paid to instruction and practice of the correct pace of delivery until students demonstrate competence.

The Processing Speed Index subtests tend to be of the lowest complexity to administer, and most of the errors in this area related to verbatim instructions. There were, however, a number of errors made with the sample item and practice items for the Symbol Search subtests, which suggests the importance of examiner familiarity with these.

The very low rate detection of errors in the self- and peer-reviews (12 and 5% respectively) compared to the faculty member review is of significant interest and concern. Previous research (e.g., Blakey et al., 1987; Kuentzel et al., 2011) has found that peer review and peer checking procedures help to improve some aspects of administration and correct some errors, but that significant errors still remain. It is likely that errors were identified by students and peers in first attempts at the video recording with the errors then corrected in the version submitted to the faculty member. Students may not recognize errors because they are novices themselves and do not have sufficient experience to detect errors, even with the use of a structured checklist. This suggests that cross-level peer reviewing (e.g., senior students or new graduates checking the work of students in their first year of a professional training program) may be a more effective strategy. Further, in the current study, the fact that the administration and scoring task was completed as a course assessment may have impacted students’ willingness to report errors in their peers’ work. Anecdotal evidence indicates that students sometimes marked a criterion as correct on the checklist but noted an error in the ‘Comments’ section. To assist to overcome this, teachers may choose to allocate a proportion of the assessment task marks to the peer review and to deduct marks from the reviewer for each error that they fail to detect.

The current study represents the first known evaluation a model of competence for teaching psychology students the latest edition of the Wechsler Adult Intelligence Scale (WAIS-IV) based on Gilmore and Campbell’s (2009) model. Most students demonstrated acceptable levels of competency (91.3%) and students achieved a 93.5% rate of correct administration and scoring items on the checklist. This study provides a detailed breakdown of the number and types of administration and scoring errors made by post-graduate psychology students according to an updated checklist designed to cover all of the objective criteria for WAIS-IV administration and scoring from the test manual. Whilst the study has the strength of a high participation rate of students from within two professional psychology specializations, the use of one cohort of students at one university who were primarily female may impact the generalizability of the findings. Further, while the students were taught by faculty uninvolved in this study, the faculty member who marked the student’s administration and scoring was the first author, who was aware of the aims of the study. The findings regarding the absolute number of errors are affected by the fact that some students were known to re-record their videos to correct errors prior to submission whilst others were aware that their submission contained minor errors. However, the pattern of errors in different aspects of administration and scoring are consistent with previous literature and the authors’ experiences with previous cohorts of students. A final important point is that Australian universities are moving to teach students to administer Wechsler tests using devices (ipads) to administer and score the tests. It is possible that this method will reduce error rates substantially, however, this remains to be established.

## Conclusion

This study demonstrates that a modification of Gilmore and Campbell (2009) model of competence is effective for teaching psychology students to administer and score the WAIS-IV. However, it is well-recognized that administering a standardized intellectual functioning assessment such as the WAIS-IV requires significant multi-tasking and there is enormous potential for errors (Loe et al., 2007). The failure of peer and self-reviews to detect the majority of the errors suggests that novice feedback (self or peers) may be ineffective to fully reduce errors, and the use of more senior peers may be preferable. It is suggested that involving senior trainees, recent graduates and/or experienced practitioners in the training of post-graduate students may have benefits for both parties, promoting a peer-learning and continuous professional development approach to the development and maintenance of skills in psychological assessment.

## Conflict of Interest Statement

The authors declare that the research was conducted in the absence of any commercial or financial relationships that could be construed as a potential conflict of interest.
