# Supplementary material for: Assessment of a model for achieving competency in administration and scoring of the WAIS-IV in post-graduate psychology students
Source: Front Psychol. 2015 May 18;6:641. doi: 10.3389/fpsyg.2015.00641 (PMC4434897; doi:10.3389/fpsyg.2015.00641)
Supplement: Supplementary file 1 [file Table_1.DOCX]

***Supplementary Material***

**Assessment of a model for achieving competency in administration and scoring of the WAIS-IV in postgraduate psychology students**

**Rachel M Roberts^1,^*, Melissa C Davis^2^**

^1^School of Psychology, University of Adelaide, Adelaide, South Australia, Australia.

^2^School of Psychology and Speech Pathology, Curtin University, Bentley, Western Australia, Australia.

*** Correspondence:** Rachel M Roberts, School of Psychology, University of Adelaide, Adelaide, 5005, South Australia, Australia.

rachel.roberts@adelaide.edu.au

1. **Supplementary Figure 1 Assessment rubric.**

To pass the assignment all assessment criteria must be met at least at an adequate level.

| **Assessment criteria** |  | **Level of performance** | | |
| --- | --- | --- | --- | --- |
|  |  | **Excellent/ good** | **Adequate** | **Not met** |
|  |  | **STUDENT DEMONSTRATES BASIC COMPETENTCY** | | **STUDENT NOT COMPETENT** |
| **Test administration** |  |  |  |  |
| Follows test manual administration and instruction |  | All instructions in manual followed. | Occasional errors in testing but not sufficient to reduce the validity of the test | Significant errors in test administration which make this administration invalid |
| Organisation and familiarity with materials |  | Student is well organised during the testing and displays proficiency and confidence in handling test materials, application of discontinue rules, prompts etc, and manages use of record sheet smoothly | Student is moderately organised, shows some sings of hesitation or uncertainty in handling materials and administering items but this does not reduce the validity of the test | Student not appropriately prepared for administration of the test. Student is disorganised, confused or fails to respond appropriately to errors or misunderstandings made by ‘client’, or fails to seek additional information when required. Likely to make the administration invalid |
| Appropriate interpersonal interaction during testing |  | ‘Client’ set at ease, manages a good pace in the assessment, speaks clearly, is pleasant and encouraging without violating administration rules | Student shows some nervousness in the interaction, make need to adjust the pace or volume of their speech a little or may make awkward remarks, but is generally pleasant and appropriate and validity of test not reduced | Inappropriate feedback to ‘client’ about progress, speaks unclearly, (mumbles, too loud, too soft, too fast), shows own discomfort in situation. Likely to make the administration invalid |
| **Record form** |  |  |  |  |
| Record form correctly filled out |  | Student has used all required parts of form and information is in correct sections | Mostly correct use of record form, with one or two minor omissions that make no significant impact to the outcome of the assessment, responses recorded verbatim | Student has not used the form correctly, multiple errors in form use, responses not recorded verbatim. Errors reduce validity of assessment |
| Scoring is correct |  | Scoring is correct, and where interpretation is required, student has made a good effort at a correct interpretation | There may be minor errors in scoring, that have no substantive impact of the outcome of the assessment | Significant errors have been made in scoring such as failing to apply discontinue rules or to count all items, poor decisions made in applying scoring rules. Errors reduce validity of assessment |
| Discrepancy and strengths and weaknesses analysis correct |  | Analysis is correct | There may be minor errors in analysis, that have no substantive impact of the outcome of the assessment | Significant errors have been made in analysis. Errors reduce validity of assessment |

1. **Supplementary Figure 2 Administration and Scoring Checklist**

**WAIS-IV Administration and Scoring Review**

**Examiner name: _______________________________________**

**Who was this review sheet completed by?**

**Peer reviewer** 🞎 **name:_______________________________________**

**Self** 🞎

**PART 1: ADMINSTRATION AND SUBTEST SCORING**

**Introducing the WAIS**

Appropriate introduction from the manual 🞎 🞎

Unsatisfactory Satisfactory

1. **Block Design**

|  | Satisfactory/  correct | Unsatisfactory/  incorrect | Comments |
| --- | --- | --- | --- |
| **From video** |  |  |  |
| 1.Introduce blocks |  |  |  |
| 2.Demonstration item |  |  |  |
| 3.Lay-out of blocks |  |  |  |
| 4.Administration of Trial 2 (if necessary) |  |  |  |
| 5.Scrambling blocks between trials |  |  |  |
| 6.Timing |  |  |  |
| 7.Reverse rule (if necessary) |  |  |  |
| 8.Discontinuation rule |  |  |  |
| **From protocol** |  |  |  |
| 9.Record completion time |  |  |  |
| 10.Draw incorrect design |  |  |  |
| 11.Indicate whether correct design |  |  |  |
| 12.Item scoring |  |  |  |
| 13.Total Raw score |  |  |  |

1. **Similarities**

|  | Satisfactory/  correct | Unsatisfactory/  incorrect | Comments |
| --- | --- | --- | --- |
| **From video** |  |  |  |
| 1.General directions |  |  |  |
| 2.Use of queries (if necessary |  |  |  |
| 3.Reverse rule (if necessary) |  |  |  |
| 4.Discontinuation rule |  |  |  |
| **From protocol** |  |  |  |
| 5.Record responses  verbatim |  |  |  |
| 6.Item scoring |  |  |  |
| 7.Total Raw score |  |  |  |

1. **Digit Span (Forwards, Backwards, Sequencing)**

|  | Satisfactory/  correct | Unsatisfactory/  incorrect | Comments |
| --- | --- | --- | --- |
| **From video** |  |  |  |
| 1.Item instructions |  |  |  |
| 2.Pace of digits (1 per second) |  |  |  |
| 3.Discontinuation rule |  |  |  |
| **From protocol** |  |  |  |
| 4.Record responses |  |  |  |
| 5.Item scoring |  |  |  |
| 6.Total Raw score |  |  |  |

**4. Matrix Reasoning**

|  | Satisfactory/  correct | Unsatisfactory/  incorrect | Comments |
| --- | --- | --- | --- |
| **From video** |  |  |  |
| 1.General directions |  |  |  |
| 2.Item instructions |  |  |  |
| 3.Reverse rule (if necessary) |  |  |  |
| 4.Discontinuation rule |  |  |  |
| **From protocol** |  |  |  |
| 5.Record responses |  |  |  |
| 6.Item scoring |  |  |  |
| 7.Total Raw score |  |  |  |

**5. Vocabulary**

|  | Satisfactory/  correct | Unsatisfactory/  incorrect | Comments |
| --- | --- | --- | --- |
| **From video** |  |  |  |
| 1.General directions |  |  |  |
| 2.Use of queries (if necessary |  |  |  |
| 3.Reverse rule (if necessary) |  |  |  |
| 4.Discontinuation rule |  |  |  |
| **From protocol** |  |  |  |
| 5.Record responses verbatim |  |  |  |
| 6.Item scoring |  |  |  |
| 7.Total Raw score |  |  |  |

**6. Arithmetic**

|  | Satisfactory/  correct | Unsatisfactory/  incorrect | Comments |
| --- | --- | --- | --- |
| **From video** |  |  |  |
| 1.General directions |  |  |  |
| 2.Timing |  |  |  |
| 3.Reverse rule (if necessary) |  |  |  |
| 4.Discontinuation rule |  |  |  |
| **From protocol** |  |  |  |
| 5.Record completion time |  |  |  |
| 6.Record response |  |  |  |
| 7.Item scoring |  |  |  |
| 8.Total Raw score |  |  |  |

**7. Symbol Search**

|  | Satisfactory/  correct | Unsatisfactory/  incorrect | Comments |
| --- | --- | --- | --- |
| **From video** |  |  |  |
| 1.General directions |  |  |  |
| 2.Item instructions |  |  |  |
| 3.Sample items |  |  |  |
| 4.Practice items |  |  |  |
| 5.Timing |  |  |  |
| **From protocol** |  |  |  |
| 6.Record completion time |  |  |  |
| 7.Number correct/incorrect |  |  |  |
| 8.Total raw score |  |  |  |

**8. Visual Puzzles**

|  | Satisfactory/  correct | Unsatisfactory/  incorrect | Comments |
| --- | --- | --- | --- |
| **From video** |  |  |  |
| 1.General directions |  |  |  |
| 2.Timing |  |  |  |
| 3.Reverse rule (if necessary) |  |  |  |
| 4.Discontinuation rule |  |  |  |
| **From protocol** |  |  |  |
| 5.Record completion time |  |  |  |
| 6.Record response |  |  |  |
| 7.Item scoring |  |  |  |
| 8.Total Raw score |  |  |  |

**9.**  **Information**

|  | Satisfactory/  correct | Unsatisfactory/  incorrect | Comments |
| --- | --- | --- | --- |
| **From video** |  |  |  |
| 1.General directions |  |  |  |
| 2.Use of queries (if necessary |  |  |  |
| 3.Reverse rule (if necessary) |  |  |  |
| 4.Discontinuation rule |  |  |  |
| **From protocol** |  |  |  |
| 5.Record responses |  |  |  |
| 6.Item scoring |  |  |  |
| 7.Total Raw score |  |  |  |

**10. Coding**

|  | Satisfactory/  correct | Unsatisfactory/  incorrect | Comments |
| --- | --- | --- | --- |
| **From video** |  |  |  |
| 1.Correct Form |  |  |  |
| 2.General directions |  |  |  |
| 3.Item instructions |  |  |  |
| 4.Timing |  |  |  |
| **From protocol** |  |  |  |
| 5.Record completion time |  |  |  |
| 6.Total raw score |  |  |  |

**11. Letter-Number Sequencing**

|  | Satisfactory/  correct | Unsatisfactory/  incorrect | Comments |
| --- | --- | --- | --- |
| **From video** |  |  |  |
| 1.Item instructions |  |  |  |
| 2.Sample items |  |  |  |
| 3.Corrections/prompts (if necessary) |  |  |  |
| 4.Discontinuation rule |  |  |  |
| **From protocol** |  |  |  |
| 5.Record responses |  |  |  |
| 6.Item score |  |  |  |
| 7.Total raw score |  |  |  |

**12. Figure Weights**

|  | Satisfactory/  correct | Unsatisfactory/  incorrect | Comments |
| --- | --- | --- | --- |
| **From video** |  |  |  |
| 1.General directions |  |  |  |
| 2.Timing |  |  |  |
| 3.Reverse rule (if necessary) |  |  |  |
| 4.Discontinuation rule |  |  |  |
| **From protocol** |  |  |  |
| 5.Record completion time |  |  |  |
| 6.Record response |  |  |  |
| 7.Item scoring |  |  |  |
| 8.Total Raw score |  |  |  |

**13. Comprehension**

|  | Satisfactory/  correct | Unsatisfactory/  incorrect | Comments |
| --- | --- | --- | --- |
| **From video** |  |  |  |
| 1.General directions |  |  |  |
| 2.Use of queries (if necessary |  |  |  |
| 3.Reverse rule (if necessary) |  |  |  |
| 4.Discontinuation rule |  |  |  |
| **From protocol** |  |  |  |
| 5.Record responses |  |  |  |
| 6.Item scoring |  |  |  |
| 7.Total Raw score |  |  |  |

**14. Cancellation**

|  | Satisfactory/  correct | Unsatisfactory/  incorrect | Comments |
| --- | --- | --- | --- |
| **From video** |  |  |  |
| 1.General directions |  |  |  |
| 2.Demonstration item |  |  |  |
| 3.Sample item |  |  |  |
| 4.Timing |  |  |  |
| **From protocol** |  |  |  |
| 5.Record Completion time |  |  |  |
| Item 1 |  |  |  |
| Item 2 |  |  |  |
| 6.Record number correct/incorrect |  |  |  |
| Item 1 |  |  |  |
| Item 2 |  |  |  |
| 7.Item score |  |  |  |
| Item 1 |  |  |  |
| Item 2 |  |  |  |
| 8.Total Raw Score |  |  |  |

| **9.**Record responses |  |  |  |
| --- | --- | --- | --- |
| 10.Item scoring |  |  |  |
| 11.Total Raw score |  |  |  |

**15. Picture Completion**

|  | Satisfactory/  correct | Unsatisfactory/  incorrect | Comments |
| --- | --- | --- | --- |
| **From video** |  |  |  |
| 1.General directions |  |  |  |
| 2.Use of queries (if necessary |  |  |  |
| 3.Timing |  |  |  |
| 4.Reverse rule (if necessary) |  |  |  |
| 5.Discontinuation rule |  |  |  |
| **From protocol** |  |  |  |
| 6.Record responses (verbal and/or point) |  |  |  |
| 7.Item scoring |  |  |  |
| 8.Total Raw score |  |  |  |

**PART II - SCORE CONVERSION AND PROCESS ANALYSIS**

|  | **Correct** | **Incorrect** |
| --- | --- | --- |
| **1.Age calculation** |  |  |
| **2.Scaled scores** |  |  |
| **Sums of Scaled Scores** |  |  |
| 3.Verbal Comprehension |  |  |
| 4.Perceptual Reasoning |  |  |
| 5.Working Memory |  |  |
| 6.Processing Speed |  |  |
| 7.Full Scale IQ |  |  |
| **IQ/Index Scores** |  |  |
| 8.Verbal Comprehension |  |  |
| 9.Perceptual Reasoning |  |  |
| 10.Working Memory |  |  |
| 11.Processing Speed |  |  |
| 12.Full Scale IQ |  |  |
| **13.Discrepancy Comparisons** |  |  |
| **14.Strengths and Weaknesses** |  |  |
| **15.Process Analysis** |  |  |
| **16.Substitute subtest/s** used if appropriate |  |  |
